# Optimization of Planting Concrete Thickness and Grass Species for Roadbed Side Slopes

**DOI:** 10.3390/ma18204694

**Published:** 2025-10-13

**Authors:** Yuancheng Zhuang, Jun Chen, Wanxue Xu

**Affiliations:** College of Civil and Transportation Engineering, Hohai University, Nanjing 210098, China; 221304060007@hhu.edu.cn (Y.Z.); 19966593505@163.com (W.X.)

**Keywords:** planting concrete, roadbed side slope, optimal mix proportion, grass species selection, thickness optimization

## Abstract

Planting concrete is a composite material used for erosion control on roadbed side slopes. However, excessive concrete thickness creates an unfavorable environment that prevents the survival of some grass species. This study aims to optimize the thickness and grass species of planting concrete. The stress scenarios of planting concrete, including pedestrian loads and frost heave stress, were analyzed. The maximum internal stress under pedestrian loads and the frost heave stress during freezing were determined using finite element analysis and frost heave tests, respectively. Nine groups of planting concrete specimens with different porosities and water–cement ratios were prepared and tested. The measured compressive and splitting tensile strengths were compared with the maximum stress of planting concrete to determine the optimal mix proportion. Using the optimal mix, planting concrete specimens with three thicknesses were prepared, and six common grass species were selected for planting experiments. Vegetation coverage, plant height, root length, root number, and root biomass were measured for each grass species at three thicknesses to determine the optimal thickness and grass species. The results show that the maximum tensile stress of planting concrete under pedestrian loads and frost heave stress is 0.86 MPa. The optimal porosity and water–cement ratio are determined to be 30% and 0.33, respectively. Ryegrass exhibits the highest vegetation coverage and plant height, thereby determining that ryegrass is the optimal grass species. Planting concrete of 4 cm thickness demonstrates the best root development, thereby determining that 4 cm is the optimal thickness. These findings provide a scientific basis for optimizing ecological slope protection with planting concrete.

## 1. Introduction

Ecological protection of roadbed side slopes has become an essential measure to mitigate soil erosion and enhance environmental sustainability. Among the commonly applied approaches, two main techniques are employed: grass planting and planting concrete [1,2]. Grass planting provides several benefits, including effective erosion control, ecological improvement, and enhanced driving safety [3,4]. Nevertheless, its effectiveness is often constrained by rainfall-induced surface erosion, which may wash away seeds and lead to reduced retention and poor establishment [5,6]. Moreover, insufficient grass coverage can result in surface runoff channels, thereby accelerating soil loss and potentially compromising slope stability [7].

**Figure 1:**
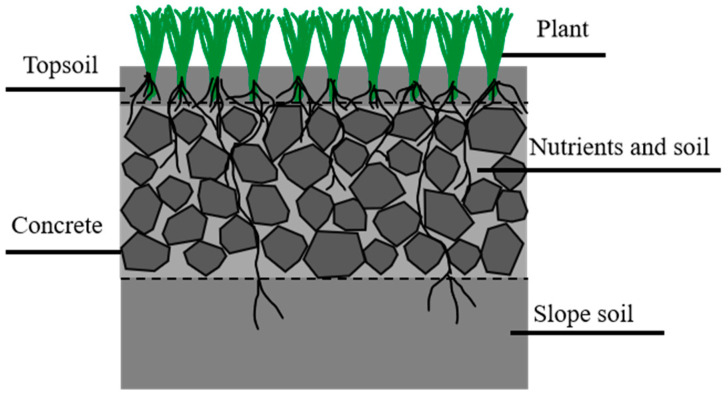
Structural configuration of planting concrete.

Planting concrete is a composite material that combines concrete with plants [8]. It is composed of vegetative soil, cement, aggregates, nutrients, water, and plant seeds [9]. The structural configuration is shown in Figure 1. The concrete matrix provides protection that enhances the survival rate of grass seeds [10]. When the seeds germinate and grow into turf, they enhance resistance to rainfall-induced erosion and contribute to the suppression of surface runoff, thereby improving the stability of roadbed side slopes [11,12]. In addition, the greening effect and ecological functions of plant roots promote enhanced slope aesthetics and biodiversity [13].

Currently, the thickness design of planting concrete is largely based on experience, with the typical design thickness being more than 10 cm [14]. However, planting concrete on roadbed side slopes is mainly subjected to pedestrian loads and freeze–thaw cycles rather than vehicular loads [15]. It is unclear whether a thickness exceeding 10 cm is appropriate under such low loads. Most previous studies have focused on mix proportion optimization, permeability, and vegetation establishment, while systematic investigations on the suitable thickness of planting concrete have been scarce [16]. Excessive thickness causes a substantial proportion of grass roots to remain within the pore soil of the planting concrete, delaying their penetration into the underlying slope soil [17]. This condition may also necessitate alkalinity reduction measures to ensure plant growth. In addition, a thicker planting concrete consumes more coarse aggregates, leading to reduced resource efficiency and increased material costs [18,19].

In addition to material thickness, the selection of appropriate grass species affects the performance of planting concrete [20]. Six grass species commonly used in planting concrete include Cynodon dactylon, Bermuda grass, broadleaf grass, white clover, ryegrass, and tall fescue. These grass species differ in root penetration ability and growth potential, and it remains unclear which species can rapidly establish roots and grow in relatively thin planting concrete [21,22]. Fast-growing grass species with well-developed roots can more rapidly penetrate the concrete pore soil and reach the underlying slope, forming a stable vegetative cover [23]. In contrast, slower-growing or weaker-rooted grass species tend to remain confined within the concrete pores and exhibit lower early-stage water and nutrient uptake, potentially compromising slope greening and the long-term effectiveness of ecological slope protection [24]. Therefore, it is necessary to determine the most suitable grass species for effective growth in relatively thin planting concrete.

Recent research on planting concrete has primarily focused on its material composition and ecological performance. While these studies highlight increasing interest in planting concrete as an eco-friendly material, most have emphasized mix design, plant adaptability, or durability improvement. In contrast, limited attention has been paid to the role of planting concrete thickness, which is a critical factor influencing both structural stability and vegetation growth. Furthermore, the combined effects of concrete thickness and grass species selection remain largely unexplored. Therefore, this study seeks to determine the optimal thickness of planting concrete for slope protection and the most suitable grass species under this optimized thickness. Unlike previous studies, our approach integrates structural stress analysis with vegetation adaptability, thereby providing a novel and comprehensive framework for optimizing planting concrete in thin-layer applications and offering practical guidance for ecological slope engineering.

## 2. Objective and Scope

This study aims to optimize the thickness and grass species of planting concrete on roadbed side slopes. There are three parts to the study. In the first part, the maximum internal stress of planting concrete under pedestrian loads and the frost heave stress during freezing were determined using finite element analysis and frost heave tests, respectively. In the second part, nine concrete mixes with different porosities and water–cement ratios were prepared and tested, and the measured compressive and splitting tensile strengths were compared with the calculated maximum internal stress to determine the optimal mix. In the third part, planting concrete specimens of three thicknesses were prepared using the optimal mix, and six common grass species were selected for planting experiments. Vegetation coverage, plant height, root length, root number, and root biomass were measured to determine the optimal thickness and most suitable grass species.

## 3. Internal Stress Analysis of Planting Concrete

Planting concrete on roadbed side slopes is primarily subjected to external environmental stresses, including pedestrian loads during maintenance and frost heave stress under low temperatures. In this study, finite element simulation was used to calculate the internal stress under pedestrian loads, and frost heave tests were conducted to calculate the frost heave stress during freezing. Other dynamic or cyclic loads, such as wind, animal crossings, or seismic events, were not included due to their limited relevance or difficulty in quantification in this context.

### 3.1. Working Conditions of Planting Concrete on Roadbed Side Slopes

In this study, a macroscopic finite element model of the roadbed slope with planting concrete was established. The model consists of two layers: the slope soil layer and the planting concrete layer. The planting concrete was modeled as a linear elastic material, while the slope soil was represented using the Mohr–Coulomb model. An eight-node three-dimensional solid element, suitable for concrete analysis, was employed. To ensure that all deformations occurred within the modeled domain and to prevent unlimited displacement in any direction, displacement constraints were applied at the boundaries.

**Figure 2:**
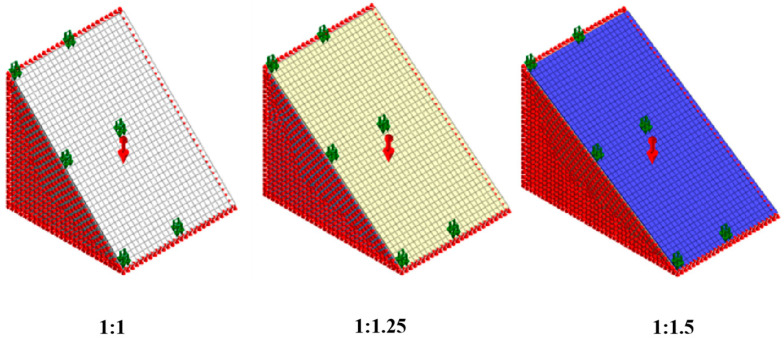
Finite element models of planting concrete on roadbed side slopes.

To calculate the maximum internal stress of planting concrete on roadbed side slopes under pedestrian loads, four factors were considered: slope gradient, soil modulus, concrete modulus, and concrete thickness. A multi-condition finite element model was constructed for simulation, with a total of 81 combined working conditions, as shown in Table 1. Finite element models of planting concrete under three typical slope gradients are shown in Figure 2.

### 3.2. Internal Stress of Planting Concrete Under Pedestrian Loads

**Figure 3:**
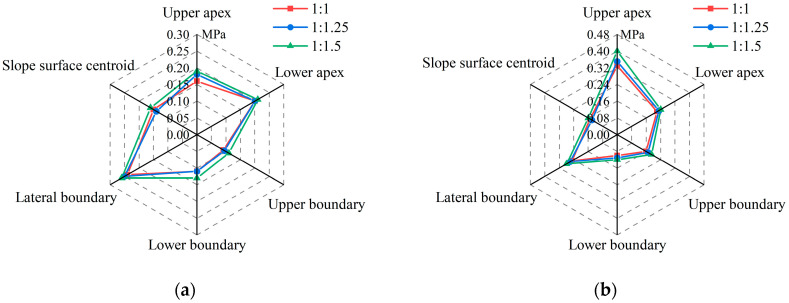
Maximum internal stresses at six load application points: (**a**) Maximum tensile stresses; (**b**) maximum compressive stresses.

When planting concrete is applied to roadbed side slopes, its internal stresses vary depending on the location subjected to pedestrian loads. Six load application points were considered in this study: (a) upper apex, (b) lower apex, (c) upper boundary, (d) lower boundary, (e) side boundary, and (f) slope surface centroid. Uniform loads with an area of 0.04 m^2^ and an intensity of 17.5 kN/m^2^ were applied independently at these locations. Simulation results indicate that the maximum internal stress occurs when the slope gradient is 1:1.5, the modulus of soil is 40 MPa, the modulus of concrete is 35 GPa, and the concrete thickness is 4 cm. The specific tensile and compressive stresses are shown in Figure 3.

As shown in Figure 3, the compressive stresses within the concrete are consistently higher than the tensile stresses. The magnitude of these stresses varies with both the load application position and the slope gradient. As the slope gradient decreases, both compressive and tensile stresses increase. This may be attributed to the increased bending moment arm in gentler slopes, which leads to higher tensile strain at the tension-critical lateral edge. The maximum tensile stress of 0.26 MPa occurs at the lateral boundary, and the highest compressive stress of 0.40 MPa is observed at the upper apex.

### 3.3. Internal Frost Heave Stress of Planting Concrete

When the temperature drops below freezing, pore water within planting concrete freezes and expands, generating frost heave stress. If this stress exceeds the cohesive strength of the concrete matrix, it can propagate existing microcracks and initiate new ones, eventually leading to surface cracking. Thus, considering the effects of frost heave stress is essential for evaluating the performance of planting concrete in cold environments.

**Figure 4:**
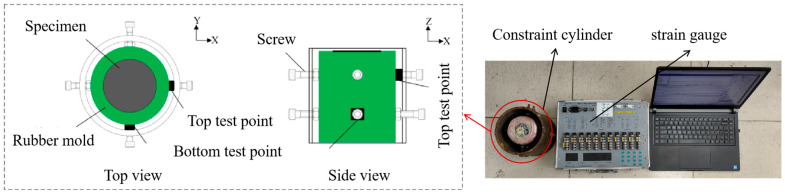
Schematic diagram of frost heave stress testing device [25].

In this study, a custom-designed frost heave stress testing device was developed to measure the internal stresses generated during concrete freezing. Figure 4 shows a schematic diagram of the device, which comprises three main components: a constraint cylinder, a strain gauge, and a data acquisition system. The concrete specimen is placed within the constraint cylinder, with earth pressure sensors and securing screws positioned between the outer surface of the specimen and the cylinder wall. This setup allows simultaneous measurement of stress evolution at different heights during the freezing process.

**Figure 5:**
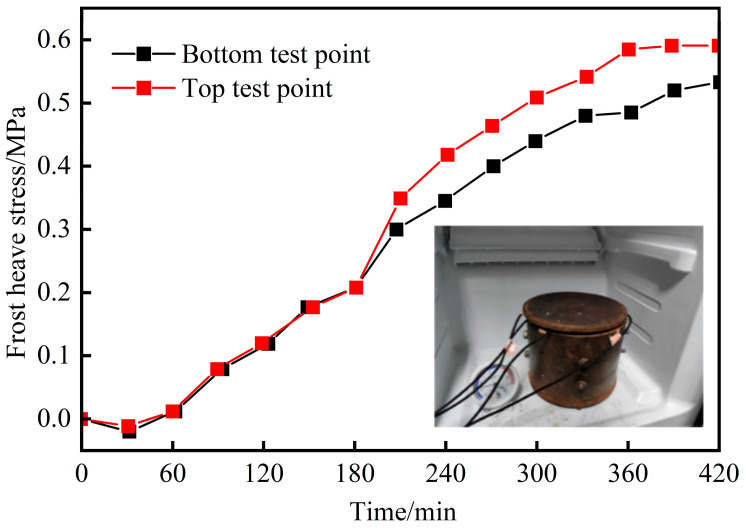
Evolution of frost heave stress in concrete specimens during freezing.

Frost heave stress testing was conducted using the device described above, with the results shown in Figure 5. At the initial freezing stage, rapid cooling caused specimen contraction, producing a transient negative stress. As freezing progressed, the internal frost heave stress gradually transitioned from negative to positive, increasing until stabilization. The stress ultimately stabilized at a peak of 0.6 MPa.

In conclusion, planting concrete is subjected to both pedestrian loads and frost heave stress. To ensure the reliable mechanical performance of planting concrete, the combined effect of these factors should be considered. Specifically, the maximum tensile and compressive stresses of planting concrete under pedestrian loads are 0.26 MPa and 0.40 MPa, respectively, and the maximum frost heave stress of the planting concrete reaches 0.6 MPa. Accordingly, the tensile and compressive strength of planting concrete should not be lower than 0.86 MPa and 1.00 MPa, respectively.

## 4. Mix Proportion Design of Planting Concrete

Owing to the unique structure of planting concrete, its mechanical properties are influenced by the mix proportion, and the mechanical performance varies with different mix proportions. In this study, based on the maximum internal stress calculated in the previous section, the compressive and splitting tensile strengths of specimens were measured to determine the optimal mix proportion.

### 4.1. Materials and Specimen Preparation

Portland cement (P.O. 42.5), crushed basalt aggregates, water, and water reducer were used to prepare planting concrete specimens. The primary properties of used cement, water reducer, and aggregate are summarized in Table 2, Table 3 and Table 4, respectively, in accordance with the CJJ/T 135-2009 [26]. The mix proportions of the planting concrete materials are shown in Table 5. It is noted that the aggregate used in this study was coarse aggregate with a particle size of 10–30 mm.

Nine groups of cement concrete specimens were prepared with water–cement ratios of 0.30, 0.33, and 0.36, and porosities of 30%, 33%, and 36%, respectively, according to the CJJ/T 253-2016 [27]. The preparation procedure was as follows:

- Aggregates, water, cement, and water reducer were weighed based on the designed mix ratios. Basalt aggregates and 50% of the total water were added to the mixer and blended for 30 s to achieve a saturated surface-dry condition;
- Cement and water reducer were then incorporated and mixed for 40 s. The remaining water was added, followed by an additional 50 s of mixing;
- The mixture was placed into cube molds in three equal layers. Each layer was tamped 15–20 times along the mold edges with a slender rod. After surface leveling, a load plate was positioned on top. The specimens were compacted using a handheld vibrator (50 Hz, 1.0 mm amplitude) for 45 s.

**Figure 6:**
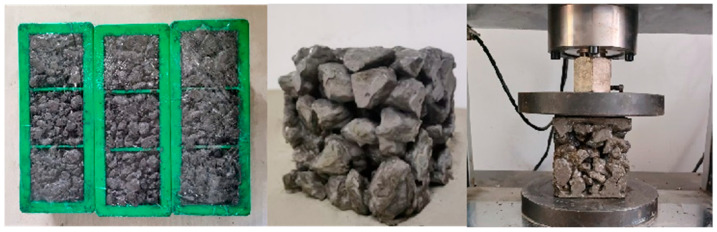
Molds and specimens prepared for mechanical tests.

All specimens were cast in 10 cm cubic molds, as shown in Figure 6. After vibration, the specimens were placed in a curing chamber maintained at 20 °C and 96% relative humidity. Specimens were demolded after 48 h and subsequently cured under standard conditions for 28 days.

### 4.2. Mechanical Strength Tests of Planting Concrete

Based on the results of finite element simulation and frost heave stress tests, the maximum tensile and compressive strengths of planting concrete specimens should reach 0.86 MPa and 1.00 MPa, respectively. In this study, following the GB/T 50081-2019 [28], the compressive and splitting tensile strengths of nine groups of specimens were tested, and the optimal mix proportion of planting concrete was determined.

#### 4.2.1. Compressive Strength Test of Planting Concrete

**Figure 7:**
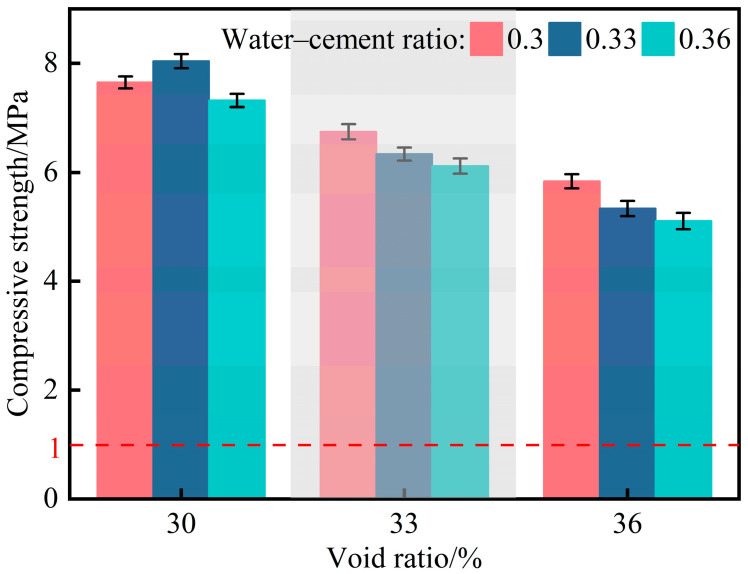
Compressive strength of nine planting concrete mixes.

Nine groups of planting concrete specimens were individually placed on the lower platen of a universal testing machine (UTM), with each specimen aligned to the center of the platen. The upper platen was adjusted to ensure it remained level and parallel to the specimen edges. Compressive strength tests were performed at a constant loading rate of 0.05 MPa/s. The compressive strength test results of the nine groups are shown in Figure 7.

As shown in Figure 7, the compressive strengths of nine groups all meet the minimum required value. The highest compressive strength was obtained with a porosity of 30% and a water–cement ratio of 0.33. At this porosity level, the compressive strength initially increased and then decreased with increasing water–cement ratio. This trend can be attributed to the relatively higher cement content under low-porosity conditions, where a moderate increase in the water–cement ratio facilitates more complete cement hydration. The resulting formation of denser and more stable calcium silicate hydrate (C-S-H) gel contributes significantly to strength development. However, when porosity exceeds 30%, a further increase in the water–cement ratio leads to a reduction in compressive strength.

#### 4.2.2. Splitting Tensile Strength Test of Planting Concrete

**Figure 8:**
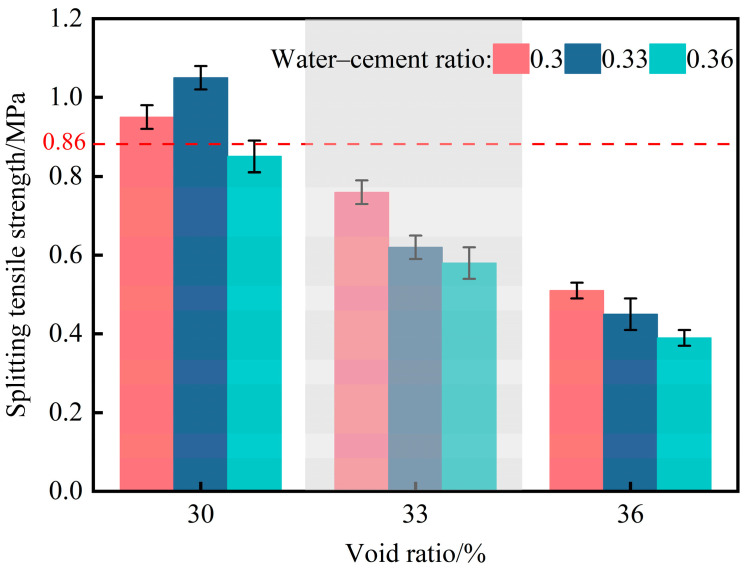
Splitting tensile strength of nine planting concrete mixes.

Considering that planting concrete on roadbed slopes is primarily subjected to indirect tensile failure, this study employed splitting tensile strength to characterize its tensile performance. The tests were conducted following the cubic splitting tensile strength method specified in the JTG 3420-2020 [29]. A linear load was applied to the opposite faces of the cubic specimens, generating a uniform tensile stress along the vertical plane at the specimen center and thereby indirectly reflecting the tensile behavior of planting concrete. The splitting tensile strength results of the nine specimen groups are shown in Figure 8.

As shown in Figure 8, only the two groups with a porosity of 30% and water–cement ratios of 0.30 and 0.33 exhibited splitting tensile strengths exceeding 0.86 MPa, achieving the minimum tensile strength. At a constant porosity of 30%, the splitting tensile strength first increased and then decreased with increasing water–cement ratio. This trend can be attributed to improved cement paste workability at moderate water–cement ratios, which enhances aggregate coating and increases paste thickness between particles, thereby improving tensile performance. However, when porosity exceeds 30%, a continuous decrease in splitting tensile strength is observed with increasing water–cement ratio.

Comprehensive analysis indicates that only the two groups with a porosity of 30% and water–cement ratios of 0.30 and 0.33 achieved the minimum compressive and tensile strengths. A comparison between these two groups shows that the 0.33 water–cement ratio specimen exhibited superior mechanical performance, with a splitting tensile strength exceeding 1 MPa. Therefore, the optimal mix design for planting concrete was determined to be a porosity of 30% and a water–cement ratio of 0.33.

## 5. Optimal Thickness and Grass Species Selection for Planting Concrete

Based on the determined optimal mix proportion, planting concrete specimens with three thicknesses were prepared, and six grass species were selected for planting experiments. The most suitable grass species and concrete thickness were determined based on the growth conditions of the planted grass species.

### 5.1. Planting Methods for Grass Species in Planting Concrete

**Figure 9:**
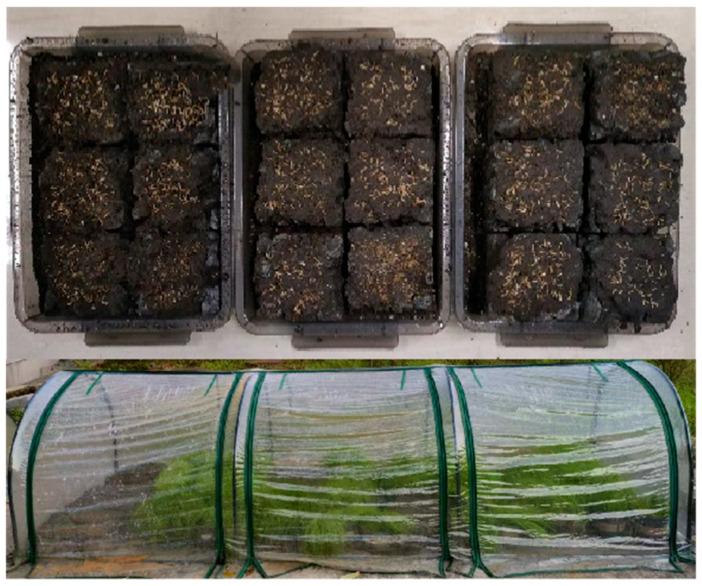
Grass seeding and growth environment.

In this study, a surface-layer planting method was adopted. Nutrient soil and regular soil were mixed at a mass ratio of 2:1, and water was added at a water–soil ratio of 1.5:1 to form a planting slurry. During grouting, the slurry was slowly poured onto the planting concrete surface to allow infiltration into the pores. Alternatively, specimens were immersed and gently agitated to remove air bubbles and ensure complete filling. Afterward, a 3 cm layer of nutrient soil was evenly applied to the surface. Grass seeds were sown at 40 g/m^2^ and then watered uniformly. To prevent seed displacement by rain and to protect the seeds from direct sunlight during early germination, all specimens were placed in a ventilated, shaded greenhouse. Temperature and humidity were maintained to match outdoor conditions, and environmental factors, including weather and temperature, were continuously monitored. Detailed information on the weather conditions can be found in Appendix A. The seeded planting concrete specimens and the growth environment are shown in Figure 9.

### 5.2. Optimal Grass Species Selection for Planting Concrete

Six native grass species were preliminarily selected considering their adaptability, stress resistance, functional performance, and ecological safety. The selection prioritized fast-growing, alkali-tolerant, and locally adapted species. The botanical characteristics of these grass species are summarized in Table 6. The optimal grass species were further selected based on key indicators, including vegetation coverage and plant height.

#### 5.2.1. Vegetation Coverage of Six Grass Species

**Figure 10:**
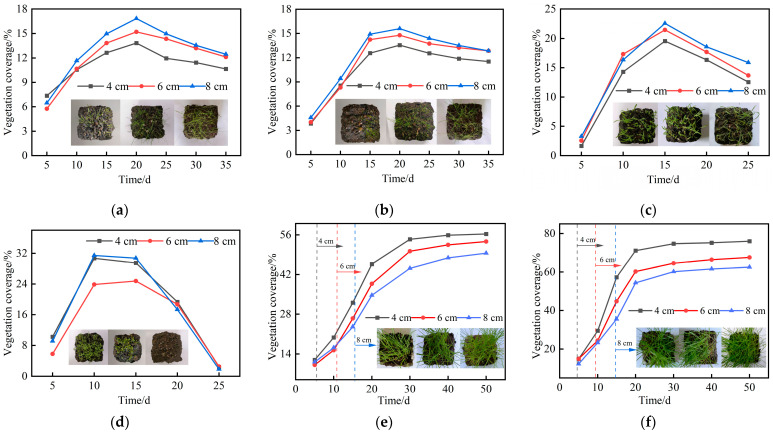
Temporal variation in vegetation coverage of six grass species: (**a**) Cynodon dactylon; (**b**) Bermudagrass; (**c**) broadleafgrass; (**d**) clover; (**e**) ryegrass; (**f**) tall fescue.

Vegetation coverage is a key indicator for evaluating plant growth performance within the planting concrete matrix, with a typical threshold value of 50% [12,30,31]. Temporal variations in coverage can be used to assess the adaptability of six grass species to the planting concrete substrate. To quantitatively analyze this dynamic process, digital images were periodically collected during the growth period. Image-Pro Plus software was used for pixel-level image processing and statistical analysis, enabling the calculation of vegetation coverage at each time point. The temporal variation of vegetation coverage for six grass species on planting concrete specimens of three thicknesses is shown in Figure 10.

As shown in Figure 10a–d, none of the four warm-season grass species achieved 50% vegetation coverage during the growth period, and all exhibited a trend of initial increase followed by decline. Among them, clover reached the highest peak coverage of 31%; however, signs of dieback were observed by day 25. No clear or consistent relationship was observed between planting concrete thickness and vegetation coverage for these species. In contrast, Figure 10e,f show that the cool-season species, ryegrass and tall fescue, both exceeded 50% vegetation coverage by day 50, with a continued upward trend thereafter. For both species, vegetation coverage decreased with increasing planting concrete thickness. Ryegrass performed best in the 4 cm specimens, reaching nearly 80% coverage by day 50. In addition, the onset of rapid coverage growth was delayed as thickness increased, occurring on day 5, day 8, and day 14 for specimens with thicknesses of 4 cm, 6 cm, and 8 cm, respectively.

#### 5.2.2. Plant Height of Six Grass Species

**Figure 11:**
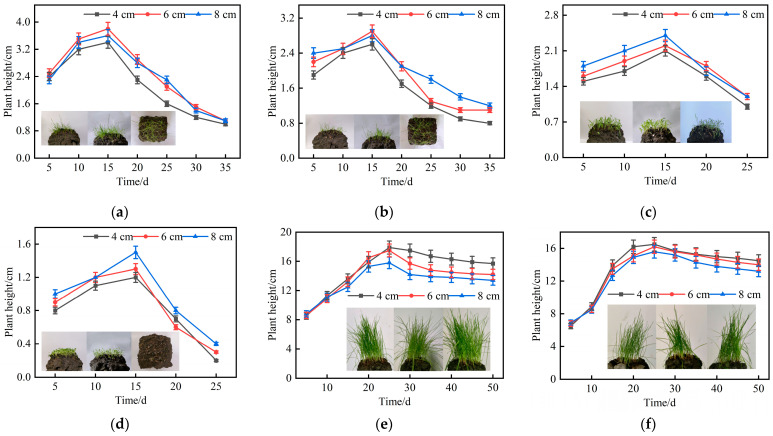
Temporal variation in plant height of six grass species: (**a**) Cynodon dactylon; (**b**) Bermudagrass; (**c**) broadleafgrass; (**d**) clover; (**e**) ryegrass; (**f**) tall fescue.

Plant height was measured at regular intervals to construct growth curves, enabling the dynamic evaluation of growth trends and species adaptability to the planting concrete substrate. The temporal variation in plant height for six grass species on planting concrete specimens of three thicknesses is shown in Figure 11.

As shown in Figure 11a–d, the four warm-season plant species reached their peak heights (ranging from 1 to 5 cm) around day 15, followed by a significant decline. No consistent correlation was observed between planting concrete thickness and the variation in plant height for these species. In contrast, Figure 11e,f show that the cool-season species, ryegrass and tall fescue, reached their maximum heights (both exceeding 10 cm) around day 25, with no significant decline in height observed in the subsequent growth stages.

In conclusion, based on the combined evaluation of vegetation coverage and plant height, the four warm-season species—Cynodon dactylon, Bermudagrass, broadleaf grass, and white clover—were deemed unsuitable for planting concrete. In contrast, the cool-season species ryegrass and tall fescue showed superior adaptability, with ryegrass outperforming tall fescue in both vegetation coverage and plant height. Therefore, ryegrass was selected as the optimal grass species for planting concrete.

### 5.3. Optimal Thickness Determination for Planting Concrete

As the primary organ responsible for water and nutrient uptake, plant roots are critical to overall growth performance. Key indicators, including root length, root number, and biomass, reflect plant health and vigor. In this study, the effect of planting concrete thickness on root development was systematically evaluated by measuring these indicators in specimens of three different thicknesses.

#### 5.3.1. Root Length of Plants

**Figure 12:**
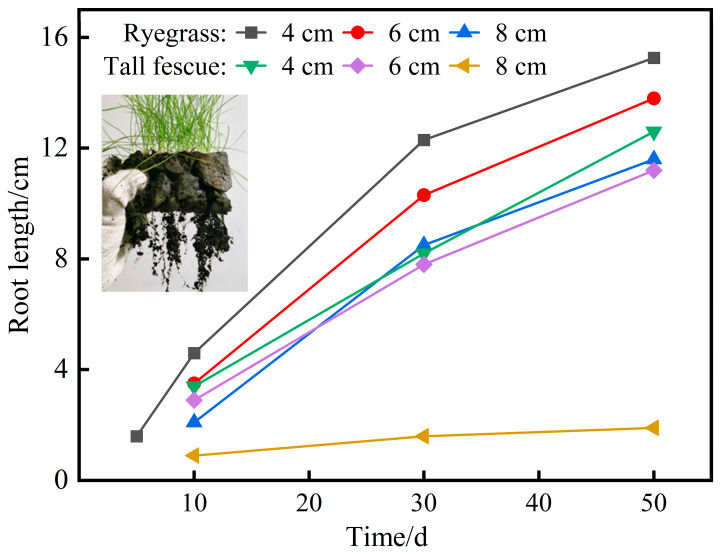
Temporal variation in root length at three planting concrete thicknesses.

During the plant growth period, photographs of the root systems were taken to monitor root development, and root lengths were measured every 20 days. Figure 12 shows the measured root lengths of ryegrass and tall fescue in planting concrete specimens with three thicknesses.

As shown in Figure 12, ryegrass exhibited the earliest root penetration in the 4 cm thick planting concrete. By day 10, both ryegrass and tall fescue had roots penetrating all three thickness levels. Root length increased rapidly during the early growth stage and then gradually plateaued. At the end of the 50-day growth period, the final root length ranking was as follows: ryegrass—4 cm > ryegrass—6 cm > tall fescue—4 cm > ryegrass—8 cm > tall fescue—6 cm > tall fescue—8 cm. Root length decreased with increasing planting concrete thickness. Notably, increasing thickness had a stronger adverse effect on tall fescue, with root penetration not exceeding 2 cm at 8 cm thickness.

#### 5.3.2. Root Number of Plants

**Figure 13:**
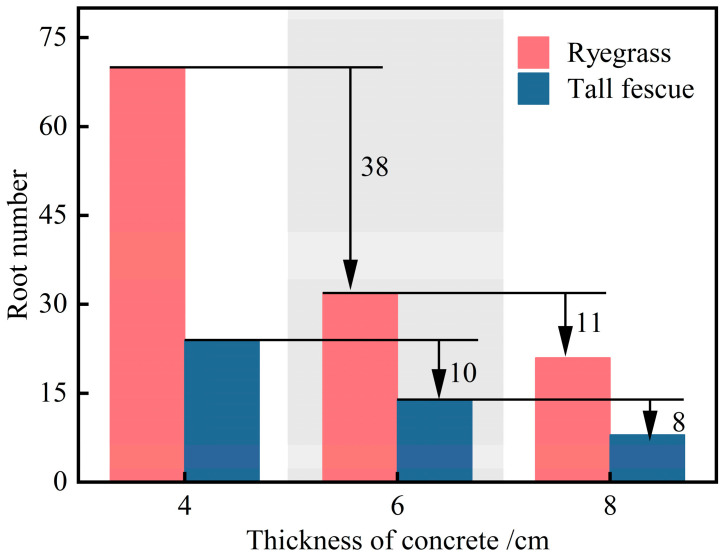
Temporal variation in root number at three planting concrete thicknesses.

After the 50-day growth period, roots were collected by cutting along the bottom of the planting concrete specimens. Due to the fine nature of the roots and to avoid the difficulty of combining roots from multiple specimens, roots were individually cut and immediately cleaned of adhering soil and impurities. This procedure ensured accurate root count statistics. The number of penetrating roots of ryegrass and tall fescue in planting concrete specimens of three thicknesses is shown in Figure 13.

As shown in Figure 13, the number of penetrating roots of both ryegrass and tall fescue decreased significantly with increasing planting concrete thickness. When the thickness increased from 4 cm to 6 cm, root numbers decreased by 38 for ryegrass and 10 for tall fescue. Further increasing the thickness from 6 cm to 8 cm reduced root numbers by 11 and 8, respectively. In the thinner range (4–6 cm), ryegrass exhibited a substantially greater reduction, indicating higher sensitivity to concrete thickness changes. However, beyond 6 cm, the inhibitory effect of increased thickness on root penetration was similarly significant for both species, suggesting a potential threshold effect.

#### 5.3.3. Root Biomass of Plants

**Figure 14:**
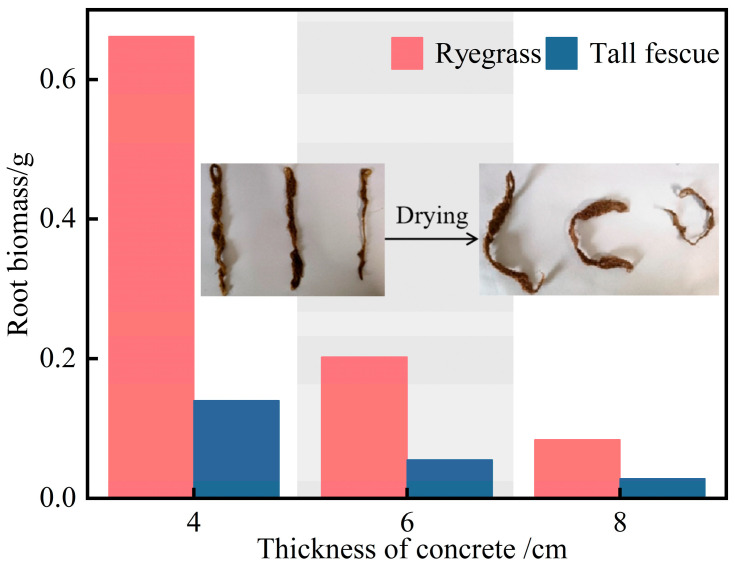
Temporal variation in root biomass at three planting concrete thicknesses.

After the 50-day growth period, roots penetrating through the bottom of the planting concrete specimens were carefully cut, washed to remove soil, and oven-dried at 50 °C until a constant weight was achieved. The dry root biomass was measured precisely with an electronic balance. Root biomass of ryegrass and tall fescue in planting concrete specimens with three thicknesses is shown in Figure 14.

As shown in Figure 14, the root biomass of both ryegrass and tall fescue decreased markedly with increasing planting concrete thickness, with the effect being more pronounced for ryegrass. At 4 cm thickness, ryegrass biomass reached approximately 0.65 g, substantially higher than tall fescue (0.15 g). At 6 cm, root biomass declined to 0.2 g for ryegrass and 0.05 g for tall fescue, and at 8 cm, both species exhibited biomass below 0.1 g. Increased planting concrete thickness inhibited root development by limiting available growth space and altering substrate pore connectivity.

Based on the root length, number, and biomass of the plants during the 50-day growth period, both grass species exhibited optimal growth in 4 cm thick planting concrete. This finding is consistent with previous studies on ecological concrete, which suggest that reduced thickness facilitates root growth by decreasing resistance and improving water and nutrient accessibility [10,24]. Accordingly, a thickness of 4 cm is recommended for planting concrete on roadbed side slopes.

## 6. Conclusions

This study analyzed the stress scenarios of planting concrete and calculated the maximum internal stresses under pedestrian loads and frost conditions using finite element simulations and frost heave tests. Nine concrete specimens with different porosities and water–cement ratios were tested to determine the optimal mix proportion. Planting experiments were then conducted with specimens of three thicknesses and six grass species to determine the optimal thickness and most suitable species. The main conclusions are summarized as follows:

(1) Planting concrete on roadbed slopes is primarily subjected to pedestrian loads and frost heave stress. To withstand these conditions, the tensile and compressive strengths of planting concrete should be at least 0.86 MPa and 1.00 MPa, respectively.
(2) Only planting concrete specimens with a porosity of 30% and water–cement ratios of 0.30 and 0.33 achieved the tensile strength of 0.86 MPa. Among these, the specimen with a water–cement ratio of 0.33 exhibited higher splitting tensile strength. Therefore, the optimal porosity and water–cement ratio are determined to be 30% and 0.33, respectively.
(3) The four warm-season species—Cynodon dactylon, Bermuda grass, broadleaf grass, and white clover—did not meet the thresholds for vegetation coverage and plant height. In contrast, ryegrass and tall fescue exhibited superior growth, with sustained coverage and normal plant height, indicating that they are the most suitable species for planting concrete.
(4) Ryegrass exhibited superior root development in 4 cm thick planting concrete, with average root length, number of penetrating roots, and biomass at day 50 exceeding those in the 6 cm and 8 cm specimens. In contrast, tall fescue showed restricted root growth and biomass in 8 cm thick concrete, indicating that 4 cm is the optimal thickness for planting concrete.

This study provides insights into the optimization of planting concrete thickness and grass species selection. However, several limitations should be acknowledged. First, the planting experiments were conducted over a short period of 50 days, which may not fully capture long-term vegetation sustainability. Second, only six grass species were tested; other species or mixed planting strategies may exhibit different growth patterns or ecological suitability. Finally, the experiments were conducted under controlled greenhouse conditions, whereas field factors such as variable rainfall, soil erosion, slope heterogeneity, and seasonal freeze–thaw or wet–dry cycles could significantly influence actual performance.

Future studies will build on the findings of this work by extending planting experiments under real field conditions with longer monitoring periods to better assess vegetation sustainability. A broader range of grass species, including mixed planting strategies, should be evaluated to determine optimal combinations for ecological slope protection. In addition, the durability of planting concrete under long-term freeze–thaw and wet–dry cycles should be investigated to ensure structural performance over time. Finally, the use of additives, such as fibers or supplementary cementitious materials, should be explored.

## Figures and Tables

**Table 1:** Working conditions used in the simulation.

Gradient of Side Slope	Modulus of Soil (MPa)	Modulus of Concrete (GPa)	Thickness of Concrete (cm)
1:1	40	15	4
1:1.25	60	25	8
1:1.5	85	35	12

**Table 2:** Physical properties of used cement [26].

Fineness (m^2^/kg)	Initial Setting Time (min)	Final Setting Time (min)	Compressive Strength (28 Days) (MPa)	Flexural Strength (28 Days) (MPa)
360	170	230	55.2	8.8

**Table 3:** Basic properties of used water reducer [26].

Appearance	Particle Size (mm)	Chloride Ion Content (%)	Sodium Sulfate Content (%)	Water Reduction Rate (%)
Brown powder	0.315	<0.4	<20	16–22

**Table 4:** Basic properties of used aggregate [26].

Apparent Density (kg/m^3^)	Bulk Volume Density (kg/m^3^)	Moisture Absorption Rate (%)
2941	2833	0.78

**Table 5:** Required mass of raw materials for 1 m^3^ of planting concrete.

Porosity (%)	Aggregate (kg)	Cement (kg)	Water (kg)	Water Reducer (kg)
30	1434	193	57.90	1.93
	1434	184	60.72	1.84
	1434	176	63.36	1.76
33	1434	145	43.50	1.45
	1434	138	45.54	1.38
	1434	132	47.52	1.32
36	1434	96	28.80	0.96
	1434	92	30.36	0.92
	1434	88	31.68	0.88

**Table 6:** Characteristics of grass species for planting concrete.

Species	Root System	Height (cm)	Nutrient Tolerance	Growth Season Type
Cynodon dactylon	Well-developed	10–40	Strong	Warm-season
Bermudagrass	Vigorous	10–40	Strong	Warm-season
Broadleafgrass	Shallow	5–15	Moderate	Warm-season
Clover	Shallow	10–30	Weak	Warm-season
Ryegrass	Well-developed	15–80	Relatively strong	Cool-season
Tall fescue	Well-developed	15–90	Relatively strong	Cool-season

## Data Availability

The original contributions presented in this study are included in the article. Further inquiries can be directed to the corresponding author.

## Appendix A

**Table A1:** Summary of weather conditions.

Date	Highest Temperature (°C)	Lowest Temperature (°C)	Climatic
25 May 2024	29	24	Cloudy
26 May 2024	32	20	Cloudy–Moderate rain
27 May 2024	18	13	Moderate rain–Cloudy
28 May 2024	27	16	Overcast–Cloudy
29 May 2024	27	19	Overcast
30 May 2024	21	19	Heavy rain–Thunder
31 May 2024	26	19	Foggy–Cloudy
1 June 2024	28	18	Overcast–Cloudy
2 June 2024	27	19	Overcast
3 June 2024	32	19	Cloudy–Overcast
4 June 2024	29	21	Overcast
5 June 2024	22	19	Foggy–Cloudy
6 June 2024	25	17	Overcast-Cloudy
7 June 2024	26	19	Overcast–Cloudy
8 June 2024	31	23	Overcast–Cloudy
9 June 2024	32	23	Overcast–Cloudy
10 June 2024	29	22	Overcast–Cloudy
11 June 2024	30	22	Overcast–Cloudy
12 June 2024	33	22	Foggy–Cloudy
13 June 2024	34	24	Foggy–Cloudy
14 June 2024	36	23	Overcast–Cloudy
15 June 2024	35	25	Foggy–Cloudy
16 June 2024	36	24	Cloudy
17 June 2024	33	21	Overcast–Cloudy
18 June 2024	32	23	Overcast–Thunder
19 June 2024	26	23	Light rain–Heavy rain
20 June 2024	32	25	Foggy–Thunder
21 June 2024	29	25	Heavy rain
22 June 2024	29	25	Thunder–Cloudy
23 June 2024	28	22	Foggy–Overcast
24 June 2024	29	22	Overcast–Thunder
25 June 2024	26	22	Light rain–Overcast
26 June 2024	28	22	Foggy–Overcast
28 June 2024	24	23	Heavy rain
27 June 2024	28	21	Light rain–Thunder
29 June 2024	25	24	Heavy rain–Light rain
30 June 2024	30	25	Overcast–Thunder
1 July 2024	30	26	Light rain–Heavy rain
2 July 2024	24	22	Moderate rain–Overcast
3 July 2024	31	26	Light rain–Cloudy
4 July 2024	34	26	Cloudy
5 July 2024	35	28	Cloudy
6 July 2024	34	28	Overcast–Cloudy
7 July 2024	36	27	Overcast–Cloudy
8 July 2024	35	29	Overcast–Cloudy
9 July 2024	33	25	Overcast–Heavy rain
10 July 2024	25	23	Heavy rain
11 July 2024	23	21	Heavy rain
12 July 2024	27	23	Heavy rain
13 July 2024	29	25	Light rain–Heavy rain
14 July 2024	27	24	Light rain–Thunder
15 July 2024	31	27	Overcast–Cloudy
